# Stabilization of High Internal Phase Oil-in-Water Emulsions Using “Whole” *Gracilaria lemaneiformis* Slurry

**DOI:** 10.3390/foods12183464

**Published:** 2023-09-17

**Authors:** Jinjin Li, Xiaoming Guo, Zhengqi Liu, Zhihua Yang, Chunqing Ai, Shuang Song, Beiwei Zhu

**Affiliations:** 1 Shenzhen Key Laboratory of Food Nutrition and Health, College of Chemistry and Environmental Engineering and Institute for Innovative Development of Food Industry, Shenzhen University, Shenzhen 518060, China; lijinjin@szu.edu.cn (J.L.); liuzhengqi@szu.edu.cn (Z.L.); zhubeiwei@163.com (B.Z.); 2 Shenzhen Institute of Standards and Technology, Shenzhen 518033, China; 3 National & Local Joint Engineering Laboratory for Marine Bioactive Polysaccharide Development and Application, Dalian 116034, China; acqdongying@163.com (C.A.); songs1008@163.com (S.S.)

**Keywords:** *Gracilaria lemaneiformis*, high internal phase emulsion, emulsifying properties

## Abstract

In this study, a *Gracilaria lemaneiformis* slurry (GLS) was prepared using low-energy mechanical shearing. The resulting GLS, which was rich in polysaccharides, was utilized as an effective stabilizer for oil-in-water emulsions. The microstructures and stability of the resulting emulsions were controlled by adjusting the emulsion formulations, including *Gracilaria lemaneiformis* (GL) mass concentration and oil volume fraction (φ). The optimized GL mass concentration and φ conditions yielded high internal phase emulsions (HIPEs) with gel-like textures. Moreover, the presence of exogenous Ca^2+^ resulted in bridging structures in the emulsions, enhancing their viscoelasticity and forming a robust physical barrier against droplet coalescence. Our findings highlight the effectiveness of the GLS as an emulsifier for stabilizing HIPEs. Notably, this method relies solely on physical processes, aligning with the desirability of avoiding chemical additives, particularly in the food industry.

## 1. Introduction

Particles made from polysaccharide-rich raw materials, including but not limited to grains [1,2], tubers [3,4], seeds [5,6], seaweeds, and other marine algae [7], have received considerable interest as Pickering particles [8]. These naturally derived biodegradable particles offer the advantage of being environmentally friendly and sustainable, making them a promising alternative to conventional synthetic materials for a variety of applications. However, the emulsification efficiency of plant-biomass-based particles is influenced by factors such as particle composition, size, and morphology, which can be controlled through nanosizing techniques employing either a “bottom-up” or “top-down” approach. The primary goal of the top-down approach is to reduce the size of large-structured materials to small particles by applying external mechanical disruptive forces [9]. This approach has gained significant attention due to its ability to precisely control particle size, shape, and composition [5,9]. There are two commonly used mechanical disruption techniques for achieving micro- or nanostructures: dry crushing (e.g., superfine grinding and ball milling) and wet crushing (e.g., colloid milling) [10]. Furthermore, homogenization, especially high-pressure homogenization, is widely employed to decrease the size of macroparticles to micro- or nano-size, particularly in emulsions or suspensions [11]. Each technology is suitable for different types of raw materials and has its strengths and weaknesses.

Seaweeds, also known as macroalgae, are a diverse group of marine plants that can be taxonomically classified into three main groups, namely, green, brown, and red [12,13]. These seaweeds are renowned for their substantial polysaccharide content, accompanied by minor proportions of proteins, lipids, and phenolic compounds, which contribute to their potential health benefits and diverse applications in the food industry [14]. Given the distinctive physical characteristics of seaweed, physical crushing techniques can be utilized to modify the macroscopic structure of seaweed in both dry and wet environments. This process results in the production of seaweed particles with different sizes and shapes, effectively increasing their specific surface area and exposing a larger number of functional groups. Subsequently, these modifications lead to the enhanced surface/interface properties of seaweed particles, thus broadening their potential applications.

*Gracilaria lemaneiformis* (GL) is a valuable seaweed widely cultivated in China [12]. Current research focuses on the extraction, structural characterization, and biological activity evaluation of active polysaccharides from GL. These polysaccharides exhibit antioxidant [13], anti-aging [15], and anti-tumor properties [16], as well as anti-allergic activity [17], and have shown promise in colitis treatment [18], gut microbiota balance [19,20], and blood glucose-lipid regulation [21,22]. However, a significant amount of residue is generated after extraction, necessitating further processing. An alternative strategy involves pulverizing the “whole” GL into fine particles using grinding technology. The high polysaccharide content of GL enables it to retain and absorb water, leading to increased swelling in GL materials during processing. Consequently, these swollen GL materials become more susceptible to shear and breakdown, with both soluble and insoluble components being released into the mixture. In our preliminary experiments, a GL slurry (GLS) was prepared by subjecting GL to heat treatment followed by high-speed physical shearing. The obtained GLS exhibited surface-active properties, as evidenced by a reduction in surface tension. However, the specific substances responsible for this property remain unclear. Based on the composition of GL, it is hypothesized in this study that polysaccharides, proteins, and their interactions may contribute to the interfacial activities and emulsifying properties of the GLS. To the best of our knowledge, there have been no prior investigations on the fabrication of seaweed particles with surface-active properties using a top-down approach. Therefore, the objective of this study is to utilize the GLS for stabilizing water-in-oil emulsions and evaluate their Pickering stability performance.

**Scheme 1:**
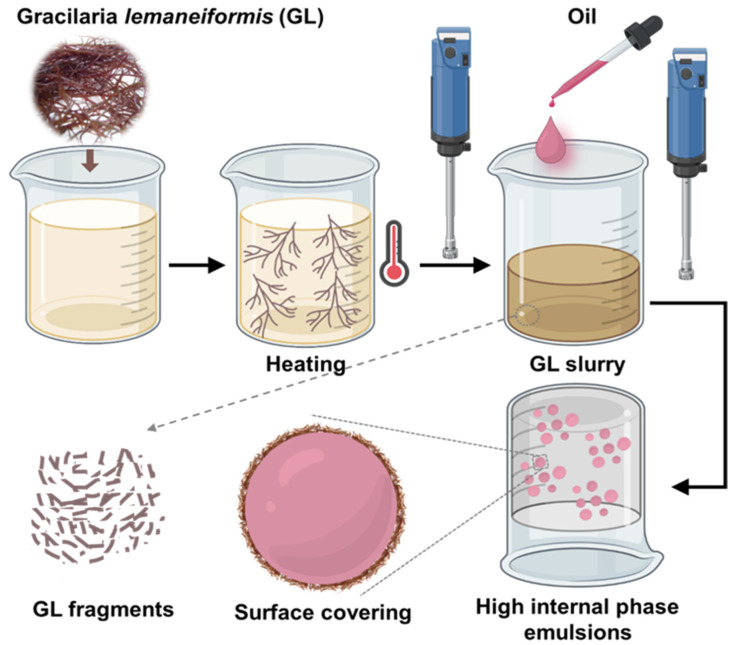
Schematic illustration of using seaweed fragments to stabilize HIPEs.

Herein, we report the preparation of high internal phase emulsions (HIPEs) using a GLS as the sole stabilizer (Scheme 1), without the addition of surfactants. Furthermore, we investigated the effects of pH levels, ionic strength, and calcium ion concentration on the characteristics of the HIPEs. Overall, this research presents a novel method for utilizing the GLS as an emulsifier and highlights the potential applications of these HIPEs in the food industry.

## 2. Materials and Methods

### 2.1. Materials

GL was the dried product from San Jie Mei Company (Fujian, China). Medium-chain triglyceride (MCT) was a product from Britz Networks Sdn. Bhd (Melaka, Malaysia). NaCl (AR, 99.5%) and CaCl_2_ (AR, 99%) were purchased from Shanghai Macklin Biochemical Co., Ltd. (Shanghai, China). Unless otherwise specified, all chemical reagents used were of analytical grade.

### 2.2. Preparation of GLS

GL samples weighing 0.25, 0.5, 0.75, 1, 1.25, 1.5, and 1.75 g were individually placed in beakers. Subsequently, 49.75, 49.5, 49.25, 49, 48.75, 48.5, and 48.25 mL of pure water were added to each beaker. The mixtures were subjected to a water bath at 80 °C for 30 min and then homogenized using a T18 homogenizer (IKA, Staufen, Germany) at 10,000 rpm and 25 °C for 3 min to make the 0.5, 1, 1.5, 2.0, 2.5, 3, and 3.5 wt % of GLS.

To prepare different pH levels of GLS, the 3 wt % GLS was prepared using the same method as described above. A GL concentration of 3 wt % was chosen based on the relatively favorable emulsification outcomes observed in our preliminary experiments. Subsequently, the pH of the GLS was individually adjusted to 3, 5, 7, 9, and 11 [23,24] using 0.1 mol/L NaOH and 1 mol/L HCl. The unadjusted pH group was designated as the control group for comparison.

The effect of ionic strength on GLS emulsification ability was investigated by adding varying amounts of powdered NaCl or CaCl_2_ salts to freshly prepared GLS samples. Specifically, NaCl powders were added to each solution to achieve ionic strengths of 0.0, 0.1, 0.3, 0.5, 0.7, and 0.9 mol/L, respectively. For GLS samples containing Ca^2+^, CaCl_2_ powders were added to each suspension to achieve Ca^2+^ concentrations of 0, 10, 20, 50, 100, 200, and 250 mmol/L.

### 2.3. Structural Characterization

#### 2.3.1. Compositional Determination

The determination of the total sugars by the phenol-sulfuric acid method at an absorbance of 490 nm [25]. The total fiber content was determined using an enzymatic gravimetric approach [26,27]. The protein content of GL japonica was determined by the Kjeldahl method (N × 6.25).

#### 2.3.2. Contact Angle Analysis and Interfacial Tension

The contact angle and interfacial tension were conducted using an LSA200 drop shape analyzer (Lauda Scientific, Germany). Freeze-dried samples were compacted into pellets with a thickness of approximately 1 mm and a diameter of 10 mm. Each pellet was then immersed in an optical glass cuvette filled with MCT. A droplet of ultrapure water (10 μL) was carefully placed on the surface of the pellet. Subsequently, the image of the droplet was captured using a high-speed video camera. The three-phase contact angle was analyzed using the Surface Meter software (version 1.2.2.16, Lauda Scientific, Lauda-Königshofen, Germany). A pendant drop technique, as described in our previous study [28], was employed to evaluate the dynamic interfacial tensions at the oil–water interface of GLS, the soluble fraction derived from GL (SGL), and the residue fraction derived from GL (RGL). To investigate adsorption at the oil–water interface, droplets of the sample solution (12 μL) were formed in an optical glass cuvette filled with MCT. All measurements were performed at 25 °C, and the values provided are the average of three measurements.

#### 2.3.3. Fourier Transform Infrared Spectroscopy and Scanning Electron Microscopy

Fourier transform infrared spectroscopy (FTIR) spectra were recorded using an FTIR spectrometer (PerkinElmer, Waltham, MA, USA) in the range of 400 to 4000 cm^−1^ with a resolution of 1 cm^−1^. The method described by Ullah et al. [29] was followed, and a total of 32 scans were collected.

For scanning electron microscopy (SEM) observation, GLS, SGL, and RGL were freeze-dried for 72 h and stored in a desiccator. Prior to imaging with a Phenom Pro SEM (Thermo Scientific, Waltham, MA, USA), a thin conductive Au coating was applied to the samples after lyophilization.

### 2.4. HIPE Preparation and Characterization

#### 2.4.1. HIPE Preparation

An amount of 10 mL of GLS prepared from each group was transferred into a 100 mL beaker. To achieve oil volume fraction (φ) levels ranging from 0.1 to 0.83, a certain volume fraction of MCT was added accordingly. The emulsification process was conducted using a T18 homogenizer (IKA, Germany) at 10,000 rpm and 25 °C for 4 min. Each experimental group was replicated three times independently.

#### 2.4.2. Droplet Size and Zeta-Potential

The droplet size and droplet size distributions of the HIPEs were measured by a laser particle sizer (Bettersize 2600, Dandong, China). The refractive indexes of MCT and water were 1.470 and 1.330, respectively, as reported by Lin et al. [4]. The zeta-potential of the HIPEs was measured using a zeta potentiometer (Nano Brook Omni, Brookhaven, New York, NY, USA). Prior to measurement, each emulsion was diluted 50 times, and three replicates were taken for each measurement.

#### 2.4.3. Visual and Light Microscopy Observation

Each group of HIPEs was visually assessed by placing them in separate 10 mL transparent glass bottles. Photographs were taken to capture the appearance of the emulsions. For light microscopy observation, 20 μL of the HIPEs was taken on a slide, covered with a cover slide, and examined under appropriate magnification using an ECLIPSE Si microscopy (Nikon, Tokyo, Japan).

#### 2.4.4. Rheological Characteristics

The apparent viscosity of the emulsion was measured using a rheometer (Discovery HR-2; TA Instrument, New Castle, DE, USA). For the determination of apparent viscosity, the instrument parameters were set as follows: fixture diameter of 40 mm, clearance of 1 mm, a test temperature of 25 °C, and a shear rate range of 0.1 to 100 s^−1^. For viscoelasticity determination, the instrument parameters were set to a scanning frequency of 0.1–100 rad/s and a fixed strain of 1% [31].

#### 2.4.5. Emulsion Stability

The stability of the emulsions was evaluated using a dispersion analyzer (LUMiSizer 651, Berlin, Germany). The instrument parameters were set as follows: the number of bars was 900, the time interval was 15 s, the speed was 2120 rpm, the temperature was 25 °C, and the wavelength was 850 nm [30].

### 2.5. Statistical Analysis

Statistical analysis of the data was conducted using the SPSS software (version 27.0), employing one-way analysis of variance (ANOVA). Significant differences between treatments were determined using Duncan’s test (*p* < 0.05, n = 3).

## 3. Results and Discussion

### 3.1. Chemical Compositions

As shown in Table 1, the protein, polysaccharide, and total fiber contents of GL were 4.63 wt %, 18.2 wt %, and 15.66 wt % (wet weight basis), respectively, with the three components summing up to approximately 40%, which is consistent with the findings reported in the literature [32]. A slurry was formed by shearing the GL mixture, from which a supernatant (SGL) and a precipitate (RGL) were isolated via centrifugation (10,000 rpm × 20 min). Analyses showed that polysaccharide and protein materials left in the SGL accounted for 0.22 ± 0.02% and 0.25 ± 0.02%, respectively. This finding indicates that partial solubilization of cell wall materials occurs during the shearing process. After the removal of SGL, protein, polysaccharide, and fiber materials retained in RGL were measured to be 1.83 wt %, 10.91 wt %, and 10.56 wt %, respectively.

Extensive research has been conducted to characterize the structure of GL polysaccharides. Previous studies have shown that galactopyranose residues within GL polysaccharides can undergo partial sulfation [15,33]. The presence of sulfate groups imparts a negative surface charge to GL polysaccharides, facilitating their crosslinking with Ca^2+^. It is possible that a higher degree of sulfation results in a greater density of negative charges on the surface of GL polysaccharides. In our study, it is conceivable that soluble polysaccharides in GLS may exist in a partially sulfated form. However, quantification of the degree of sulfation of polysaccharide in GLS necessitates further measurements.

### 3.2. Morphological Properties of GL

**Figure 1:**
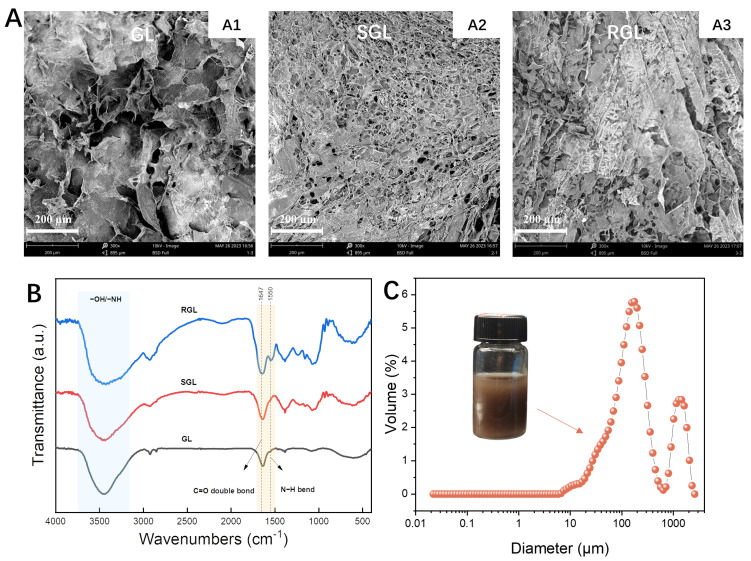
(**A**) Scanning electron micrographs of GL, SGL, and RGL. (**B**) FTIR and (**C**) droplet size distribution of the GLS (3 wt %).

As illustrated in Figure 1A, the GL sample, subjected to heating and shearing, displayed a porous and loose texture when observed under scanning electron microscopy (SEM). The unique microstructure of GL is closely associated with its rich content of polysaccharides and fibers. The presence of a porous and loose texture in GL is likely to enhance its water absorption capacity, thereby leading to a swollen appearance.

Notably, the two sub-components obtained from the initial “whole” GL slurry, namely, SGL and RGL, exhibited distinct morphological structures. SGL exhibited a relatively dense, uneven, and honeycomb-like structure, while RGL showed large sheet-like structures with smooth and fragmented surfaces. A comparison between SGL and RGL highlights the structural changes in GL resulting from the combined heating and shearing treatment.

### 3.3. FTIR Analysis

Figure 1B presents the FTIR spectra obtained for GL, SGL, and RGL. In this section, particular attention was focused on characteristic bands: in the 3750–3300 cm^−1^ region, all spectra obtained for the GL samples analyzed in the present study had a characteristic peak at 3450 cm^−1^ due to the expansion and contraction vibration of O-H and N-H, the 2900 cm^−1^ peak was attributed to C-H stretching, and the two bands at about 1647 cm^−1^ and 1550 cm^−1^ were assigned to the amide Ⅰ and amide Ⅱ protein vibrations [34,35]. Highly specific to amino acids and proteins was the peak at 1390 cm^−1^, where C-H deformation vibration overlapped the C-N stretching in amide III; the peaks from 1180 to 890 cm^−1^ corresponded to C-O stretching in carbohydrates [36]. These characteristic peaks exhibited similarities to those reported for red algae [34]. Comparison of GL with the other two samples revealed that the intensity of the peak at 1550 cm^−1^ in RGL was significantly increased, and the peaks at 1380 cm^−1^ and 1180–890 cm^−1^ in RGL and SGL were significantly enhanced. This result suggests that shearing may expose proteins and carbohydrates and make functional groups more prominent. Additionally, the presence of characteristic absorption peaks assigned to amide I, amide II, and amide III protein vibrations at approximately 1647 cm^−1^, 1550 cm^−1^, and 1390 cm^−1^, respectively, provides strong evidence for the existence of proteins within GLS. These protein vibrations are highly specific to amino acids and proteins, which indirectly supports the hypothesis that proteins may contribute to the emulsification activity of GLS.

### 3.4. Particle Size Distribution of GLS

Figure 1C illustrates the particle size distribution of the GL after mechanical shearing. The results demonstrated the formation of two distinct groups of particles. The majority of particles exhibited sizes ranging from 10 to 600 μm, with minor peaks observed at larger sizes ranging from 0.6 to 2.6 mm. This outcome suggests that applying low-energy mechanical shearing to the GL enables the generation of partial micrometer-sized GL fragments.

### 3.5. Surface Wettability and Interfacial Tension

**Figure 2:**
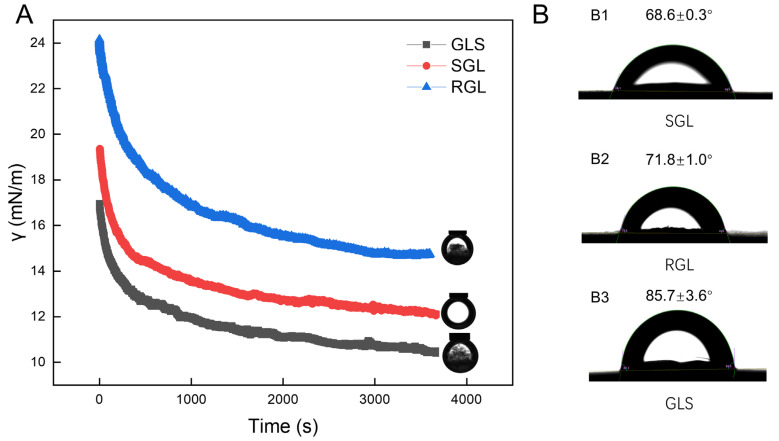
(**A**) Interfacial tension of different GL samples over time. (**B**) Contact angles of pellets prepared from lyophilized GL and its derived fractions. Data are expressed as means ± standard deviation.

As shown in Figure 2A, the interfacial tension decreased for GLS, SGL solution, and RGL dispersion, indicating that all three samples exhibited interfacial activity. The lower the interfacial tension, the higher the interfacial activity; a comparison in interfacial tension data showed that GLS was the most surface-active among the three samples. This may be attributed to the presence of polysaccharides and proteins in GLS, which have certain amphiphilic properties and are key structures that give the polymer interfacial activity [37].

As presented in Figure 2B, the contact angles of the various samples followed the order of GLS > RGL > SGL, indicating that SGL exhibited the highest surface wettability, while GLS demonstrated the lowest surface wettability. It is important to note that the contact angle of GLS was close to 90°, indicating a desirable balance between hydrophilicity and hydrophobicity [38]. This observation suggests that GLS may possess emulsion stabilizing activity.

### 3.6. Emulsifying Properties

#### 3.6.1. Effect of GL Concentration

**Figure 3:**
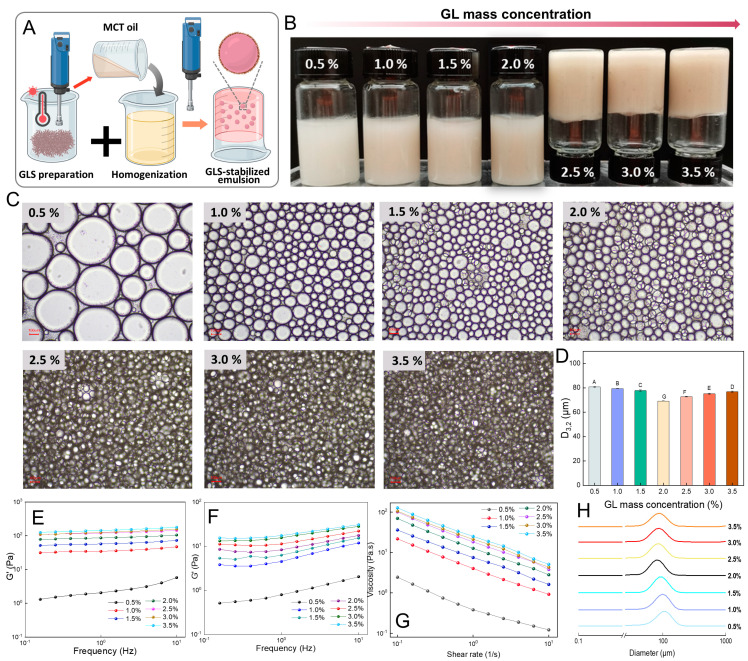
(**A**) Schematic of the preparation of GLS-stabilized HIPE. (**B**) Visual appearance of GLS-stabilized HIPEs. (**C**) Light microscopy images showcasing the oil droplets of the GLS-stabilized emulsions. (**D**) Mean droplet sizes of the GLS-stabilized HIPEs as a function of GL mass concentration; statistical differences are shown by different letters above each bar (*p* < 0.05). (**E**) Storage modulus (G′), (**F**) loss modulus (G″), (**G**) apparent viscosity, and (**H**) oil droplet size distribution of the GLS-stabilized HIPEs.

As depicted in Figure 3A, the GLS obtained through mechanical crushing was mixed with MCT oil and subsequently subjected to high-speed treatment (10,000 rpm × 4 min), resulting in the formation of a water-in-oil emulsion (Figure 3B). At lower GL mass concentrations, the GLS tends to form a coarse emulsion with droplet sizes ranging from tens to hundreds of micrometers (Figure 3C). As the GL concentration increased from 0.5 wt % to above 2.5 wt %, the droplet sizes of the resulting HIPE (φ = 0.75) decreased, and the emulsion gradually transformed from a fluid-like behavior to a gel-like state. This observation suggests that a GL concentration of 2.5 wt % represents the critical concentration required for stabilizing MCT oil. The underlying reasons for this phenomenon can be attributed to two factors: firstly, the content of interfacial surfactant species increases with an elevated GL concentration, and secondly, the rise in GL concentration enhances the viscosity of the continuous phase, providing structural support for the HIPE.

Microscopic analysis reveals that at a low GL concentration of 0.5 wt %, the volume of oil droplets within the internal phase of the emulsion was considerably large (Figure 3C). However, as the GL concentration reached 1.0 wt %, a significant reduction in observed oil droplet size was observed. Furthermore, no notable changes in oil droplet size and morphology were observed when the GL concentration ranged from 1 to 2 wt %. It should be noted that at a GL concentration of 2.5 wt %, the GL HIPE assumes a gel-like state, resulting in closely packed oil droplets. Without dilution, it is difficult to observe dispersed individual oil droplets that are difficult to observe individually without dilution. Similar results were observed with GL concentrations of 3 wt % and 3.5 wt %. The close packing of emulsion droplets may result in gel-like rheological features.

As shown in Figure 3E,F, G′ values rose along with increasing GL mass concentration. Except for 0.5 wt % GL, G′ exceeded G″, indicating the formation of gel-like structures and the predominance of elastic behavior in the emulsion’s rheological performance across the entire frequency range. Moreover, both G′ and G″ exhibited weak frequency dependence, suggesting the presence of strongly flocculated elastic structures. With increasing shear rate, the apparent viscosities of all GLS-stabilized emulsions decreased (Figure 3G). This shear thinning behavior can be attributed to the forces exerted on the emulsion structure, promoting better alignment and increased sliding between droplets, resulting in decreased viscosity. This behavior is commonly observed in various emulsions and is often desirable in applications where spreadability or flowability is required.

Data obtained from average particle size measurements reveal that the average particle diameter of the resultant GL HIPE ranged from 69.2 to 80.6 μm (Figure 3D), depending on the GL concentration. The corresponding particle size distribution curves indicate that each sample exhibits a monomodal distribution.

The findings presented in Figure 3 demonstrate that by modifying the mass concentration of GL, HIPEs with varying average sizes, viscoelastic moduli, and apparent viscosities can be obtained. Considering factors such as particle size, emulsion viscoelastic properties, and reduction of GL consumption, a concentration of 3 wt % is deemed suitable. Throughout the subsequent sections, unless explicitly stated otherwise, the GL concentration is maintained at 3 wt %.

#### 3.6.2. Effect of φ

**Figure 4:**
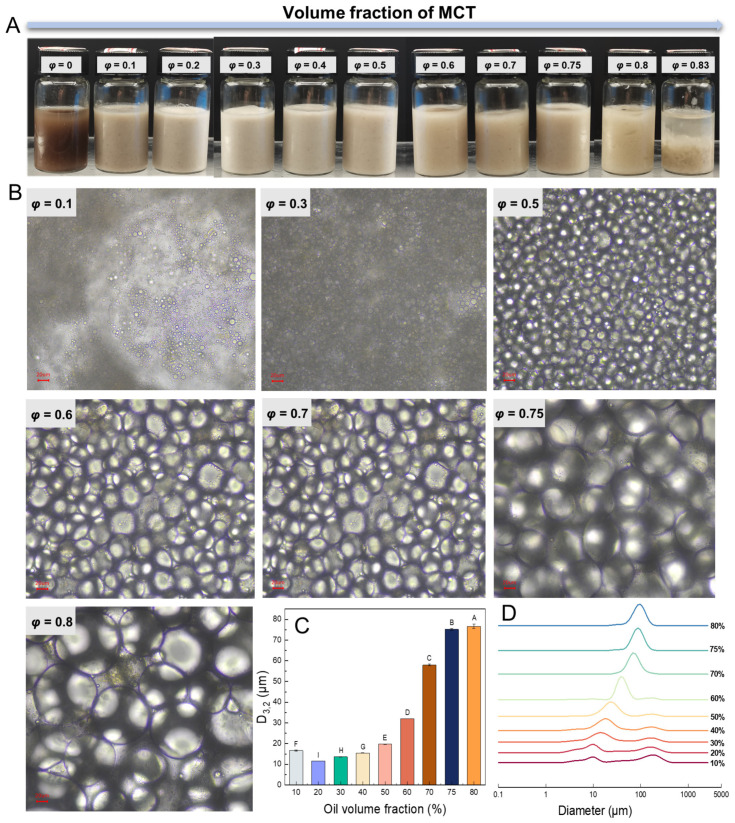
(**A**) Visual appearance of GLS and the resulting emulsions at different φ. (**B**) Light microscopic images of the GLS-stabilized emulsions prepared at φ ranging from 0.1 to 0.8. (**C**) Mean droplet sizes and (**D**) droplet size distributions of the GLS-stabilized HIPEs as a function of φ; statistical differences are shown by different letters above each bar (*p* < 0.05).

In this section, we investigated the capability of the GLS to prepare emulsions with varying φ values (Figure 4). At a mass concentration of 3 wt %, GLS was found to successfully prepare oil in water emulsions with φ ranging from 0.1 to 0.8. However, increasing φ beyond 0.83 did not yield stabilized emulsions. These results suggest that a maximum φ of 0.8 may be achievable for GLS emulsions.

Microscopic analysis of the freshly prepared emulsions revealed the presence of numerous round-shaped oil droplets with varying diameters (Figure 4B). Remarkably, it was observed that the droplet size increased progressively with an increasing φ, and within the range of 0.5–0.8, the droplets tightly packed together, forming a highly concentrated emulsion. This phenomenon can be attributed to the higher φ values, which lead to a decrease in the available space for oil droplets within the continuous phase [39]. As a result, the droplets are forced to come into proximity to one another, resulting in a more compact arrangement.

Measurement of the oil droplet characteristics revealed that mean droplet size ranged from 11.5 to 76 μm (Figure 4C) within the φ range of 0.1–0.8. However, it was not possible to measure the mean droplet size at φ = 0.83 due to the emulsion breakdown. Furthermore, considerable variation in the droplet size distribution patterns was observed among the different samples (Figure 4C). Bimodal distribution profiles were observed for the emulsions with low internal phase formed at relatively low φ values of 0.1–0.5 (Figure 4D). In contrast, the GLS-stabilized emulsions with φ values of 0.6–0.8 displayed nearly Gaussian oil droplet size distributions (Figure 4D).

#### 3.6.3. Effect of pH

**Figure 5:**
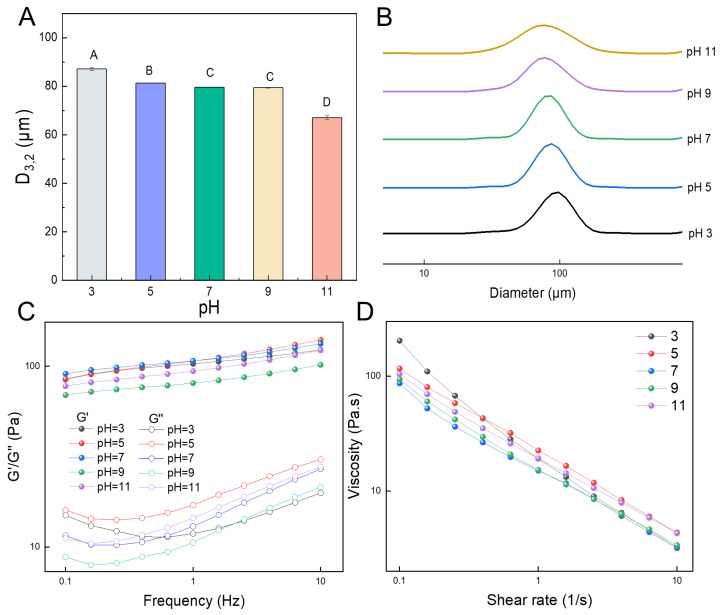
(**A**) Average particle size (D_3,2_) and (**B**) droplet size distributions of the GLS-stabilized emulsions prepared at different pH values; statistical differences are shown by different letters above each bar (*p* < 0.05). (**C**) Storage and loss moduli of the GLS-stabilized emulsions against pH. (**D**) Apparent viscosity of the GLS-stabilized emulsions prepared at different pH values.

As presented in Figure 5A, the average particle size (D_3,2_) of the GLS-stabilized emulsion exhibited a gradual decrease with increasing pH from 3 to 7. However, further increasing the pH to 9 did not yield any significant changes in the D_3,2_. A noteworthy observation was the significant decrease in D_3,2_ when the pH was increased from 9 to 11. Additionally, the particle size distributions of the GLS-stabilized emulsion prepared at different pH values (Figure 5B) exhibited similar patterns, suggesting that pH had no significant impact on the particle size distribution of the emulsion. These findings suggest that the changes in the content and structure of interfacial active substances in the GLS induced by pH may be responsible for this phenomenon.

Under alkaline conditions, proteins are more likely to dissolve from GL [40], leading to an increased concentration of interfacial active components in the solution. This can potentially enhance the emulsification efficiency of GLS. However, it is important to consider practical applications, where finding the optimal pH range is crucial to ensure effective emulsification while maintaining product stability. Factors such as safety and compatibility with other ingredients or processing conditions should also be taken into account. In the specific case of a pH level of 11, it is important to note that achieving such a high pH is uncommon in practical applications due to concerns related to product stability and safety. Therefore, although increasing the pH may theoretically improve the emulsification efficiency of GLS, it may not be practically valuable or feasible for real-world applications.

Within the tested range of frequencies, the G′ of the GLS-stabilized emulsion was consistently higher than the G″, indicating that the emulsions prepared under different pH conditions predominantly exhibited elastic behaviors (Figure 5C). A comparison of the different samples revealed that the pH 9 sample had significantly lower G′, while the emulsions obtained at pH 5 and 7 exhibited higher elastic moduli. Further investigation is needed to understand the underlying reasons for these differences in elastic modulus. Additionally, the apparent viscosities of the emulsion samples in the pH series displayed shear thinning behavior, as evidenced by the decrease in viscosity with increasing shear rate (Figure 5D).

#### 3.6.4. Effect of Na^+^

**Figure 6:**
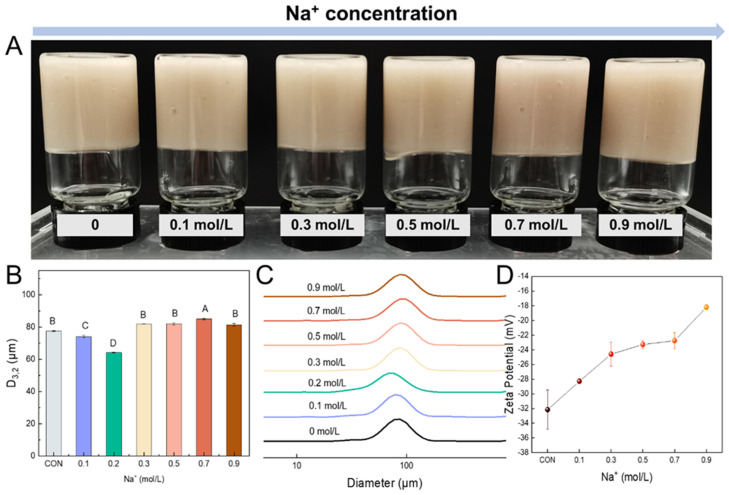
(**A**) Visual appearance of GLS-stabilized emulsions obtained at different NaCl concentrations. (**B**) Effects of NaCl concentration on D_3,2_ and particle size distribution (**C**) of the GLS-stabilized emulsions. Statistical differences are indicated by different letters above each bar, with a significance level of *p* < 0.05. (**D**) Changes of zeta-potential with NaCl concentrations ranging from 0.1 to 0.9 mol/L.

Figure 6A shows the GLS-stabilized HIPEs with different concentrations of Na^+^. The average particle size of GLS-stabilized emulsions initially decreased and then increased with increasing Na^+^ concentration (Figure 6B,C). This can be attributed to the effect of Na^+^ concentration on the dispersing system. When the Na^+^ concentration increases, two main changes may occur. On the one hand, lower concentrations of Na^+^ may partially neutralize the surface charges of the polysaccharide-based GL fragments (Figure 6D), promoting the adsorption of GL fragments onto the surface of the oil droplets and, consequently, resulting in smaller average particle sizes. On the other hand, when increasing the Na^+^ concentration above 0.3 mol/L, further neutralization of the surface charges occurred (Figure 6D). This led to increased attractive forces between the particles or droplets, ultimately causing them to aggregate into larger clusters or gel structures. Consequently, the average particle size increases.

#### 3.6.5. Effect of Ca^2+^

**Figure 7:**
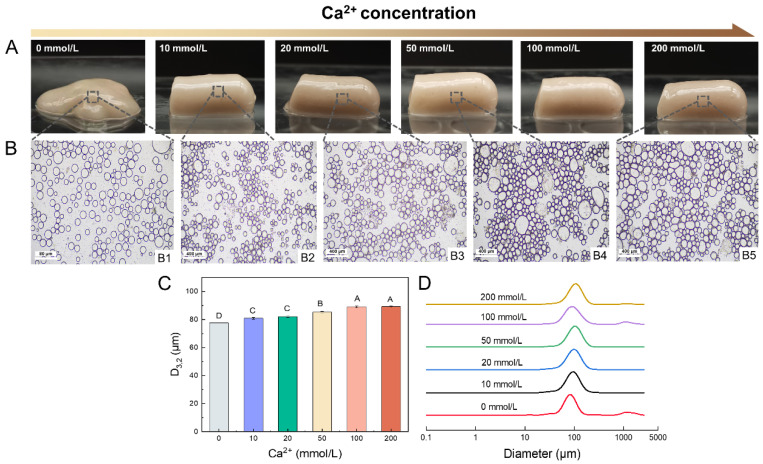
(**A**) Visual appearance of the GLS-stabilized emulsion in the presence of Ca^2+^ ranging from 0 to 200 mmol/L. (**B**) Light microscopy micrographs for the GLS-stabilized emulsion: 0 mmol/L Ca^2+^ (B1), 10 mmol/L Ca^2+^ (B2); 20 mmol/L Ca^2+^ (B3), 50 mmol/L Ca^2+^ (B4), 200 mmol/L Ca^2+^ (B5). Effect of Ca^2+^ concentration on mean droplet sizes (**C**) and droplet size distribution (**D**) for the GLS-stabilized emulsions; statistical differences are shown by different letters above each bar (*p* < 0.05).

As shown in Figure 7A, the GLS-stabilized emulsion exhibited a liquid-like behavior; it was easily pourable and unable to maintain its shape when spread on a glass slide (Figure 7A). However, the addition of Ca^2+^ at a concentration of 10 mmol/L significantly increased the gel strength of the GLS-stabilized emulsion, allowing it to form shapes and remain stable without deformation. This phenomenon was consistently observed at Ca^2+^ concentrations ranging from 20–200 mmol/L, suggesting that Ca^2+^ may induce crosslinking of macromolecules in the GLS. Notably, GL is rich in polysaccharides (Table 1), particularly carrageenan, which can undergo partial sulfation [40]. These sulfated monosaccharide units physically crosslink with Ca^2+^, resulting in gel formation [41]. Therefore, the gel formation of the GLS-stabilized emulsion in the presence of Ca^2+^ can be attributed to these interactions. Furthermore, when the sulfated monosaccharide components adsorb onto the surface of the oil droplets, Ca^2+^ can electrostatically interact with them, partially neutralizing the negative charge of the oil droplets and facilitating their aggregation [41].

As depicted in Figure 7B, the GLS-stabilized emulsion exhibited even dispersion of oil droplets in the absence of Ca^2+^. However, upon the addition of Ca^2+^, the oil droplets started to aggregate together. This observation implies that the presence of Ca^2+^ acts as a bridging agent for the oil droplets. This bridging effect may be responsible for the gradual increase in the D_3,2_ value of the emulsion as the Ca^2+^ concentration increases, as depicted in Figure 7C. Furthermore, when analyzing the particle size distribution pattern in Figure 7D, it could be observed that the presence of Ca^2+^ did not alter the shape of the distributions but rather caused a slight shift towards larger particle sizes.

### 3.7. Emulsion Stability

**Figure 8:**
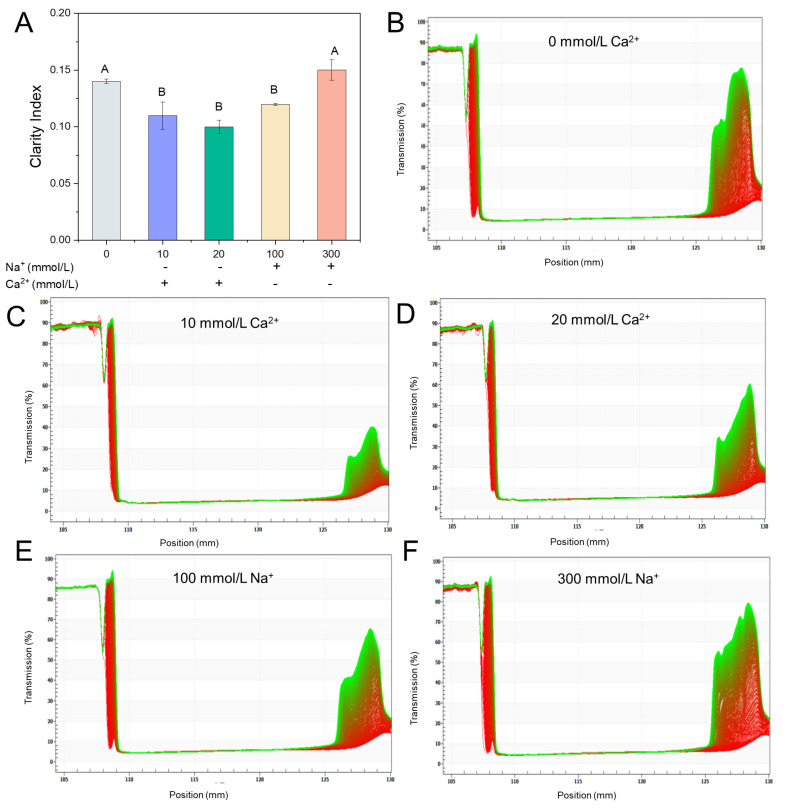
(**A**) The instability index of the GLS-stabilized emulsions; statistical differences are shown by different letters above each bar (*p* < 0.05). The light transmission curves of the GLS-stabilized emulsions: (**B**) control sample (0 mmol/L Ca^2+^ or Na^+^), (**C**) 10 mmol/L Ca^2+^, (**D**) 20 mmol/L Ca^2+^, (**E**) 100 mmol/L Na^+^, and (**F**) 300 mmol/L Na^+^.

The stability of the GLS-stabilized emulsions was assessed by determining the instability index (Figure 8A). Upon centrifugation at 25 °C, the transmission profiles of the emulsions were measured to assess the percentage of transmitted light at different positions in the cuvette, which is indicative of the creaming phenomenon. Results in Figure 8B–F demonstrate the occurrence of creaming in all tested emulsions.

Interestingly, the emulsions supplemented with 10 mmol/L Ca^2+^, 20 mmol/L Ca^2+^, and 100 mmol/L Na^+^ demonstrated significantly enhanced stability compared to the control sample, as evidenced by lower instability indices (*p* < 0.05) [42]. This improvement in stability may be attributed to the strengthened gel-like structure, which forms a steric barrier that hinders the coalescence and upward migration of oil droplets [39]. However, the addition of 300 mmol/L Na^+^ failed to improve the stability of the GLS-stabilized emulsions system. This observation may be attributed to the electrostatic shielding effect, where the surface potential of the oil droplets decreases with increasing ionic strength [41]. Consequently, the electrostatic repulsion between oil droplets is weakened, leading to their aggregation and upward movement.

## 4. Conclusions

We fabricated HIPEs by using a GLS prepared via low-energy mechanical shearing. The microstructures and stability of the GLS-stabilized emulsions were controlled by adjusting the emulsion formulations. Under optimized conditions of GL mass concentration and φ, the resulting GLS-stabilized HIPEs exhibited gel-like textures. Additionally, with the addition of exogenous Ca^2+^, these emulsions formed bridging structures, leading to enhanced viscoelasticity and a stronger physical barrier against droplet coalescence. Altogether, our findings highlight the effectiveness of the GLS as an efficient emulsifier for stabilizing HIPEs. Importantly, this method relies solely on physical processes without the use of any chemical additives, making the GLS-stabilized emulsions advantageous for food applications.

## Figures and Tables

**Table 1:** Contents of protein, total sugar, and total fiber for GL and its components * obtained before and after heating or in combination with shearing.

Sample	Protein wt %	Total Sugar wt %	Total Fiber wt %
GL **	4.63 ± 0.14 ^A^	18.20 ± 0.08 ^A^	15.66 ± 0.39 ^A^
Soluble fraction derived from GL ** (SGL)	0.22 ± 0.02 ^B^	0.25 ± 0.02 ^B^	/
Residue fraction derived from GL ** (RGL)	1.83 ± 0.08 ^C^	10.91 ± 0.01 ^C^	10.56 ± 0.25 ^B^

Different uppercase letters (A, B, and C) indicate significance between samples in the same column; data are expressed as means ± standard deviations of triplicates. ** On a wet weight basis.

## Data Availability

The data presented in this study are available upon request from the corresponding author.
